# *Lasiopodomys fuscus* as an important intermediate host for *Echinococcus multilocularis*: isolation and phylogenetic identification of the parasite

**DOI:** 10.1186/s40249-018-0409-4

**Published:** 2018-03-31

**Authors:** Qi-Gang Cai, Xiu-Min Han, Yong-Hai Yang, Xue-Yong Zhang, Li-Qing Ma, Panagiotis Karanis, Yong-Hao Hu

**Affiliations:** 1 College of Veterinary Medicine, Gansu Agricultural University, Lanzhou, 730070 China; 2 State Key Laboratory of Plateau Ecology and Agriculture, Qinghai Academy of Animal Science and Veterinary Medicine, Qinghai University, Xining, 810016 Qinghai China; 3 Qinghai Provincial People’s Hospital, Xining, 810007 Qinghai China; 4 Qinghai Institute for Endemic Disease Prevention and Control, Xining, 811602 Qinghai China

**Keywords:** *Echinococcus multilocularis*, Alveolar echinococcosis, Qinghai voles, *Lasiopodomys fuscus*, PCR, Sequencing, Phylogenetic analysis

## Abstract

**Background:**

*Echinococcus multilocularis* causes alveolar echinococcosis (AE) and is widely prevalent in Qinghai Province, China, where a number of different species have been identified as hosts. However, limited information is available on the Qinghai vole (*Lasiopodomys fuscus*), which is hyper endemic to Qinghai Province and may represent a potential intermediate host of *E. multilocularis*. Thus, *L. fuscus* could contribute to the endemicity of AE in the area.

**Methods:**

Fifty Qinghai voles were captured from Jigzhi County in Qinghai Province for the clinical identification of *E. multilocularis* infection via anatomical examination. Hydatid fluid was collected from vesicles of the livers in suspected voles and subjected to a microscopic examination and PCR assay based on the barcoding gene of *cox 1*. PCR-amplified segments were sequenced for a phylogenetic analysis. *E. multilocularis*-infected Qinghai voles were morphologically identified and subjected to a phylogenetic analysis to confirm their identities.

**Results:**

Seventeen of the 50 Qinghai voles had *E. multilocularis*-infection-like vesicles in their livers. Eleven out of the 17 Qinghai voles presented *E. multilocularis* infection, which was detected by PCR and sequencing. The phylogenetic analysis showed that all 11 positive samples belonged to the *E. multilocularis* Asian genotype. A morphological identification and phylogenetic analysis of the *E. multilocularis*-infected Qinghai voles confirmed that all captured animals were *L. fuscus*.

**Conclusions:**

*L. fuscus* can be infected with *E. multilocularis* and plays a potential role in the life cycle and epidemiology of *E. multilocularis* in the Qinghai-Tibetan Plateau of China.

**Electronic supplementary material:**

The online version of this article (10.1186/s40249-018-0409-4) contains supplementary material, which is available to authorized users.

## Multilingual abstracts

Please see Additional file 1 for translations of the abstract into the five official working languages of the United Nations.

## Background

*Echinococcus multilocularis* is a cestode species of tiny tapeworms that belongs to the *Echinococcus* genus in the Taeniidae family. To date, nine *Echinococcus* species have been reported worldwide: *E. granulosus*, *E. equinus*, *E. canadensis*, *E. felidis*, *E. ortleppi*, *E. multilocularis*, *E. oligarthra*, *E. vogeli* and *E. shiquicus* [1]. *E. multilocularis* can cause alveolar echinococcosis (AE) and is responsible for 0.3–0.5 million human AE cases in the worldwide [2, 3]. AE is one of the most dangerous zoonotic parasitic diseases and has a high fatality rate of 50–70%.

Human infection with the larval stages occurs by the ingestion of tapeworm eggs in contaminated food and water or upon contact with the final hosts. The pathogen mainly develops in the liver of the intermediate host, and liver infections are characterized by numerous vesicles, which can invade and destroy the surrounding tissues during development. In many cases, the metacestode can spread from the liver to other thoracic and/or intestinal organs [4, 5]. The incubation period of AE can vary from less than 5 years to 15 years or more. However, in most cases, patients are asymptomatic in the initial phase [6].

AE cases are geographically distributed only in the Northern Hemisphere, including North America [7], west-central Europe [8, 9], Iran [10–13], Iraq [14, 15], western and central China (Tibet Autonomous Region, west and south of Gansu Province, northwest Sichuan and southeast Qinghai Province) [16–20], Korea [21], and northern Japan, mainly in Hokkaido Island [22].

*E. multilocularis* can circulate between wild and domestic canids, which are considered definitive hosts, and other mammals, which are considered as intermediate hosts. In Qinghai Province, the Tibetan sand fox (*V. ferrilata*), red fox (*V. vulpes*), wolves, and dogs have been identified as definitive hosts that cause natural infection by *E. multilocularis* [23]. Other mammals considered the main intermediate hosts in this region include plateau pikas (*Ochotona curzoniae*), certain vole species (*Phaiomys leucurus*, *Neodon Irene* and *Microtus limnophilus*), Tibetan hares (*Lepus oiostolus*), hamsters (*Cricetulus kamensis*), and marmots and zokors [24, 25].

The high population density of rodents is linked to the high prevalence of *E. multilocularis*, and they are considered the main intermediate hosts of *E. multilocularis* and play a key role in the transmission of AE [26–30]. The living conditions of wildlife have improved under the protective activities of the Chinese government, and rodent populations have increased in number in recent years. The increased rodent populations have led to concerns among Chinese authorities over the spread of AE because of the role of these animals as the intermediate hosts for *Echinococcus* [20, 31–39]. Verifying whether a rodent species could be an intermediate host is important for recognizing the pathways of parasite transmission and controlling this disease.

**Fig. 1:**
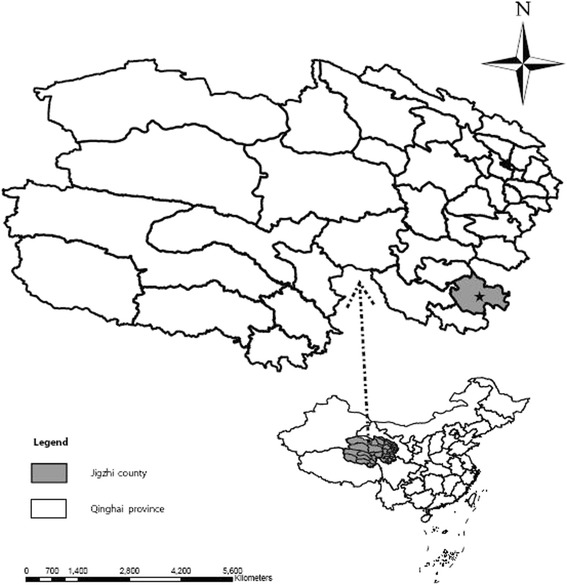
Sampling site (marked by a black star) in southeast Qinghai Province, China

Qinghai is in the northeastern part of the Qinghai-Tibet Plateau in northwest China (Fig. 1). Qinghai vole (*L. fuscus*) is a rodent species in the Cricetidae family, and it inhabits moist meadows of high mountain grassland areas, especially in the southern region of Qinghai Province. This animal is regarded as a species of least concern, and its population is unlikely to decline quickly enough to qualify for listing in a more threatened category [40–44]. This type of animal is easy and legal to catch. In this study, *E. multilocularis* was isolated from 11 Qinghai voles and subjected to PCR detection and phylogenetic analyses. The results indicated the key role of *L. fuscus* as an intermediate host in the spread of AE and transmission of *E. multilocularis* in humans and animals.

## Methods

### Sample collection

**Fig. 2:**
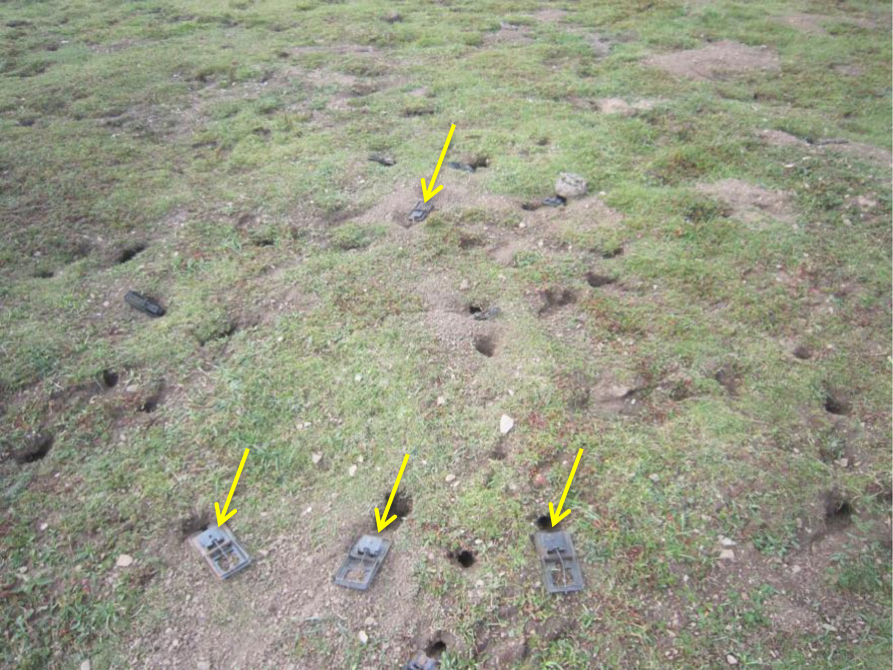
Snap trapper (yellow arrow) used to capture Qinghai voles in grassland

The sampling sites were in the southeast region of Qinghai Province and neighboring Gansu Province to the east and Sichuan Province to the south (Fig. 1). All Qinghai voles were captured from Jigzhi County in the Golog Tibetan Autonomous Prefecture (TAP) of Qinghai Province from June to September in 2015. The voles were trapped using a commercial snap trapping device (Fig. 2).

### Anatomy of the captured Qinghai voles

All of the captured Qinghai voles were dissected and examined for typical vesicle lesions in the liver. A pocket lens was used to examine the liver if typical vesicle lesions were not observed. Hydatid fluid was collected from the vesicles using a sterile syringe and kept in a low-temperature container.

### Hematoxylin-eosin (H-E) staining of vesicles

The vesicle was cut and fixed in 10% formalin for fixation. The sample was subsequently processed and embedded in paraffin. Four-micrometer-thick sections were cut and stained with hematoxylin and eosin (H-E). The H-E-stained sample slides were microscopically examined.

### Isolation and microscopic examination of protoscoleces

Fifty-milliliter tubes containing the hydatid fluid and protoscoleces were centrifuged at 1000 rpm for 5 min at 4 °C. The supernatant was discarded, and 30 ml of saline was added for dissolving and washing the sediment. The 50 ml tubes were vortexed gently and centrifuged again. The washing step was repeated 2 times. All sediment in each tube was collected into a 1.5 ml tube, and then 20 μl of the sediment was dropped onto the slides, covered with a cover slip and examined with a stereoscopic microscope for the presence of protoscoleces. The remaining protoscoleces samples were used for DNA extraction and PCR analysis.

### PCR analysis

Genomic DNA was extracted from the protoscoleces using a standard commercial kit (DNeasy Tissue Kit, Qiagen, Germany) as recommended by the manufacturer. In brief, 100 μl protoscoleces samples were incubated in lysis buffer at 55 °C for 3 h, and the genomic DNA was absorbed in a spin column. After the spin column was washed, the genomic DNA was eluted with 200 ml distilled water and used for the PCR template [45]. PCR primers, including forward (5′-TTG AAT TTG CCA CGT TTG AAT GC-3′) and reverse (5′-GAA CCT AAC GAC ATA ACA TAA TGA-3′) primers, were used to amplify part (875 bp) of the cytochrome oxidase subunit I (*cox 1*) gene [27]. The *E.* multilocularis mitochondrial genome sequence (GenBank Accession No: AB018440) was targeted.

The PCR reactions were conducted in a 50 μl (total volume) reaction mixture containing 2 μl of template DNA, 1 μl of dNTPs mix (10 mmol/L of each), 0.2 μmol/L aliquots of each primer, 0.5 U HotStar*Taq* DNA Polymerase (QIAGEN, Germany), 10 × PCR Buffer and 5 × Q-Solution. Thirty-five cycles of thermal reactions were performed, with an initial denaturation step of 15 min at 95 °C and then 94 °C for 30 s, 55 °C for 30 s and 72 °C for 60 s.

All PCR amplifications included both negative and positive controls.

### Phylogenetic analysis

The PCR-amplified segments were sequenced for a phylogenetic analysis using MEGA 7.0 software. The segments were compared with the following *cox 1* reference sequences of different *E. multilocularis* genotypes retrieved from the GenBank database: AB688127.1 to AB461417.1 for the Asian genotype, AB688134.1 to AB461413.1 for the European genotype, AB353729.1 to AB461418.1 for the North American genotype, AB510023.1 to AB510024.1 for the Mongolian genotype, and other species KP161209.1 for *E. equinus*, GQ168811.1 for *E. granulosus*, AB235846.1 for *E. ortleppi* and AB893262.1 for *E. canadensis*. A phylogenetic tree was constructed with the above sequences using the neighbor-joining method and the *p*-distance matrix for the nucleotides with the pair-wise deletion option.

### Species identification of *E. multilocularis*-infected Qinghai voles

All captured voles were examined by experts, including Professor Yuanzhong Wang from the Qinghai Institute for Endemic Disease Prevention and Control, who followed the identification key in the Qinghai Economic Animal Zoography [46].

Tail and ear tissues from *E. multilocularis*-infected Qinghai voles were used for genomic DNA extraction using a commercial kit (DNeasy Tissue Kit, Qiagen, Germany). The extracted DNA was used as a template to yield a DNA fragment (approximately 650 bp) of the *cox 1* gene by PCR using a PCR premix kit (Premix Taq™, TaKaRa). The PCR primers were VF (5’-TTC TCA ACC AAC CAC AAA GAC ATT GG-3′) and VR (5′-TAG ACT TCT GGG TGG CCA AAG AAT CA-3′) [47]. Each PCR reaction mixture (50 μl) contained 3 μl genomic DNA (approximately 10 ng), 25 μl 2 × Premix *Taq*, 1 μl each primer (10 μmol/L each) and 20 μl ddH_2_O. The PCR amplification conditions were as follows: 5 min at 94 °C; 35 cycles for 45 s at 94 °C, 45 s at 51 °C, and 45 s at 72 °C; and a final extension for 10 min at 72 °C.

PCR-positive products were subsequently sequenced and used for a phylogenetic analysis with those of *L. fuscus* (GenBank Accession No: JX962254.1, JX962258.1 and JX962265.1) and *Neodon fuscus* (GenBank Accession No: KP190276.1, KP190278.1 and KP190280.1) using MEGA 7.0 software.

## Results

### *E. multilocularis* infection in Qinghai voles

#### Sample collection

**Fig. 3:**
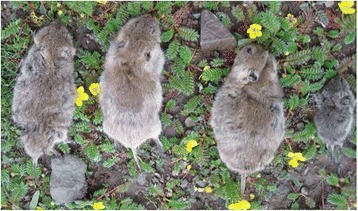
Qinghai voles captured in a grassland area

A total of 50 Qinghai voles (Fig. 3) were captured in Jigzhi County, Golog Tibetan Autonomous Prefecture of Qinghai Province.

### Anatomical characteristics of the captured Qinghai voles

**Fig. 4:**
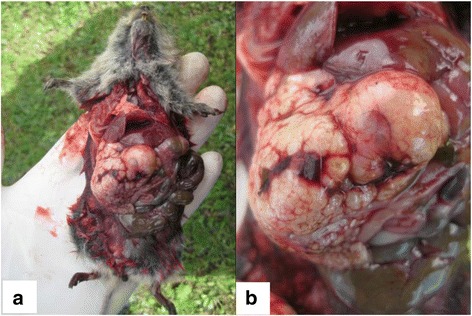
Suspected AE characterized by vesicles in the liver

Although all 50 captured Qinghai voles were checked, only 17 were found to contain AE-like vesicles in their livers (Fig. 4).

### H-E staining of vesicles

**Fig. 5:**
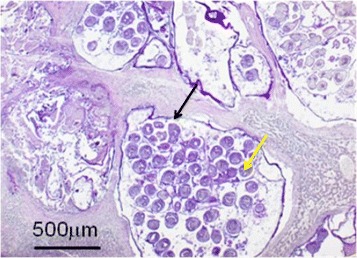
H-E staining of the vesicles and the protoscoleces (40×). The black arrow shows the entire vesicle, and the yellow arrow shows the protoscoleces in the vesicle

Vesicles and protoscoleces were stained by H-E staining. Some vesicles were empty, while others were full of protoscoleces (Fig. 5).

### Isolation and microscopic examination of protoscoleces

**Fig. 6:**
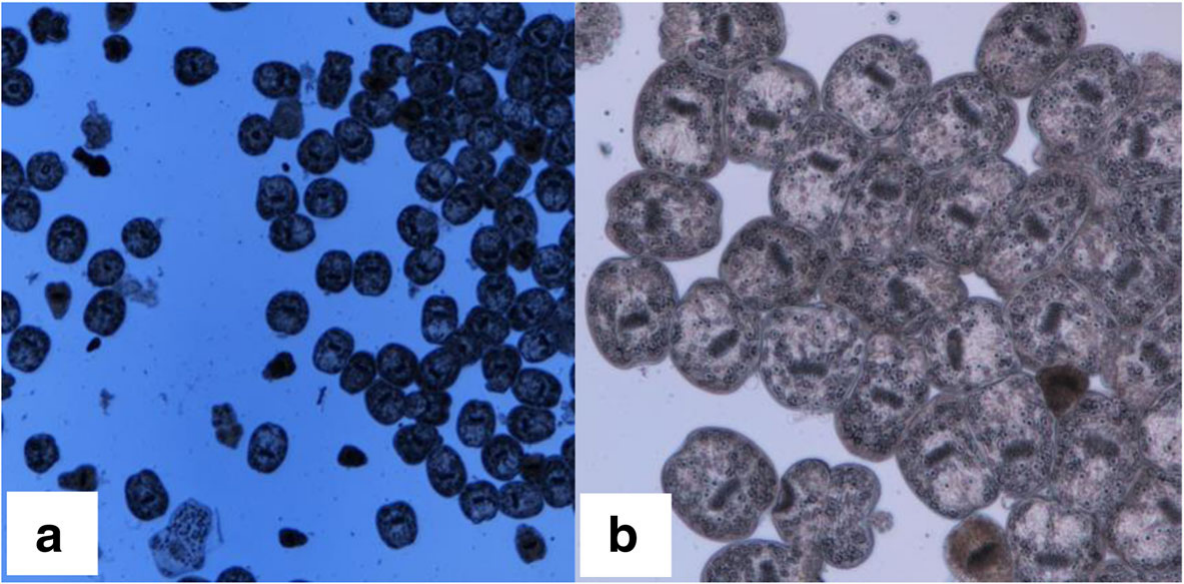
Protoscoleces examined under a stereoscopic microscope: **a** 40× and **b** 100×

After protoscoleces isolation and microscopic examination, thousands of protoscoleces were microscopically examined (Fig. 6). Eleven of 17 suspected cases were confirmed to be infected with *E. multilocularis*.

### PCR analysis

**Fig. 7:**
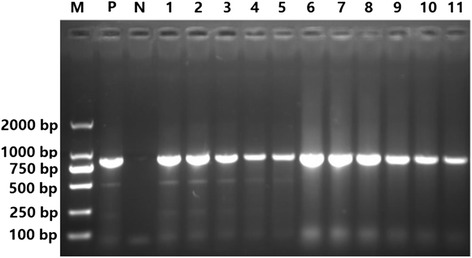
Agarose gel electrophoresis assay of the protoscoleces *cox 1* gene of *E. multilocularis*. Lane M, DL 2000 molecular marker; Lane P, Positive control; Lane N, Negative control; and Lanes 1-11: Samples of *E. multilocularis* 1 to 11

The DNA extracted from the isolated protoscoleces of the 11 samples was used as a template for amplifying the *cox 1* gene of *E. multilocularis*. PCR-positive bands were 875 bp in size. The results showed that all 11 samples were confirmed to be positive for *E. multilocularis* DNA (Fig. 7).

### Phylogenetic analysis

**Fig. 8:**
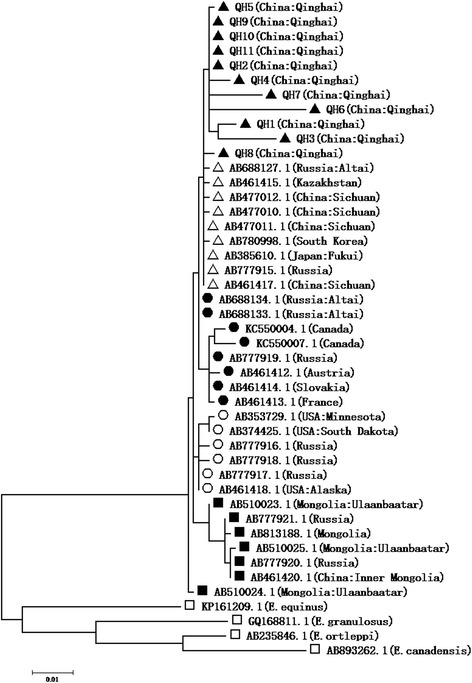
Phylogenetic comparison of the *cox1* isolated sequences with those deposited in the GenBank database. A phylogenetic tree was constructed using the neighbor-joining method and the p-distance matrix for nucleotides with the pair-wise gap deletion option. The *E. multilocularis* strains isolated from Qinghai as well as AB688127.1 to AB461417.1 belonged to the Asian genotype (marked by ▲ and △), AB688134.1 to AB461413.1 belonged to the European genotype (marked by ●), AB353729.1 to AB461418.1 belonged to the North American genotype (marked by ○), and AB510023.1 to AB510024.1 belonged to the Mongolian genotype (marked by ■); in addition, sequence KP161209.1 belonged to *E. equinus*, GQ168811.1 belonged to *E. granulosus*, AB235846.1 belonged to *E. ortleppi* and AB893262.1 belonged to *E. canadensis* (marked by □)

Purified *cox 1* products after PCR analysis were used for sequencing, and then alignment with a reference sequence (GenBank Accession No: AB477011.1) was performed. The results showed that 47 nucleotide sites were different from those of the reference sequence. The different nucleotide sites exhibited 4 characteristics (Additional file 2: Figure S1). The phylogenetic analysis showed that all positive samples belonged to the *E. multilocularis* Asian genotype (Fig. 8).

### Species identification *of E. multilocularis*-infected Qinghai voles

**Fig. 9:**
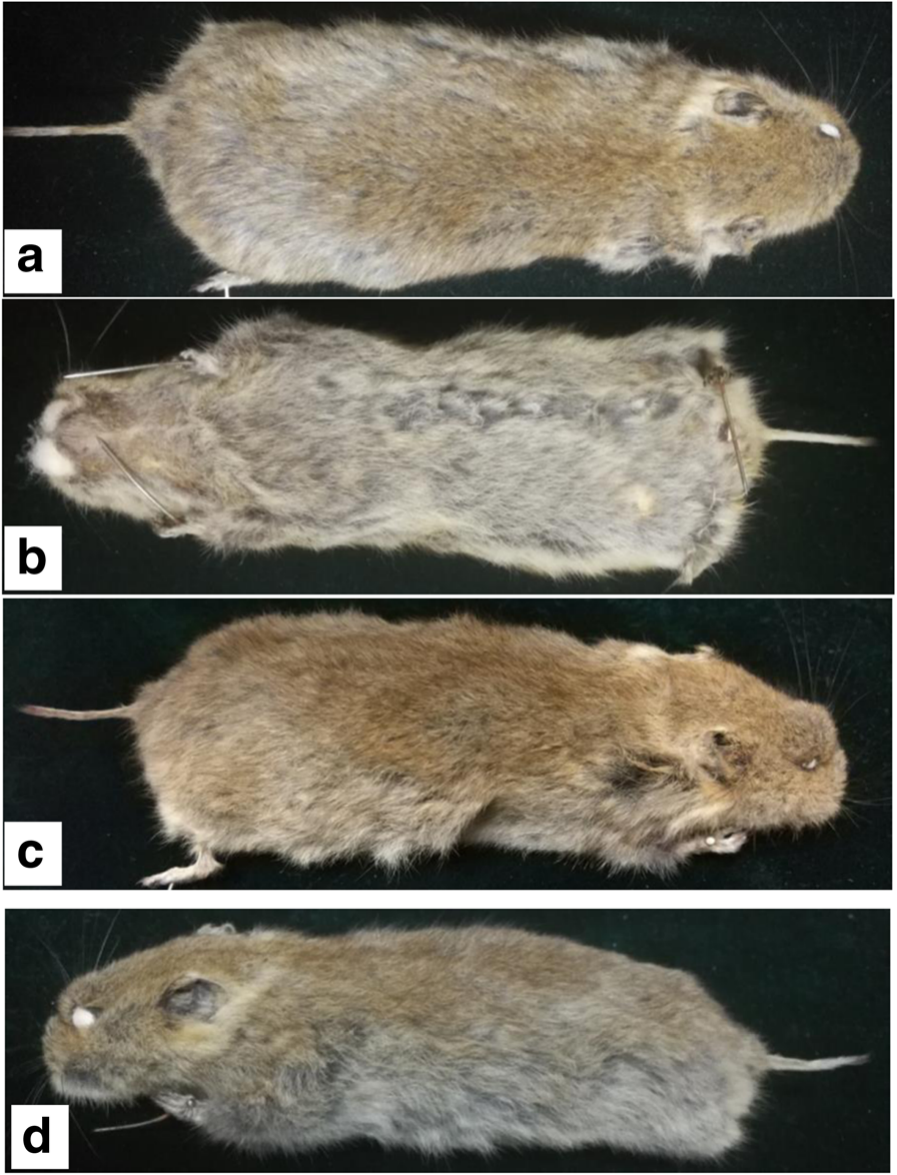
Morphological characteristics of Qinghai voles from different aspects: **a** Dorsal view; **b** Ventral view; and **c** Right lateral view; **d** Left lateral view

**Fig. 10:**
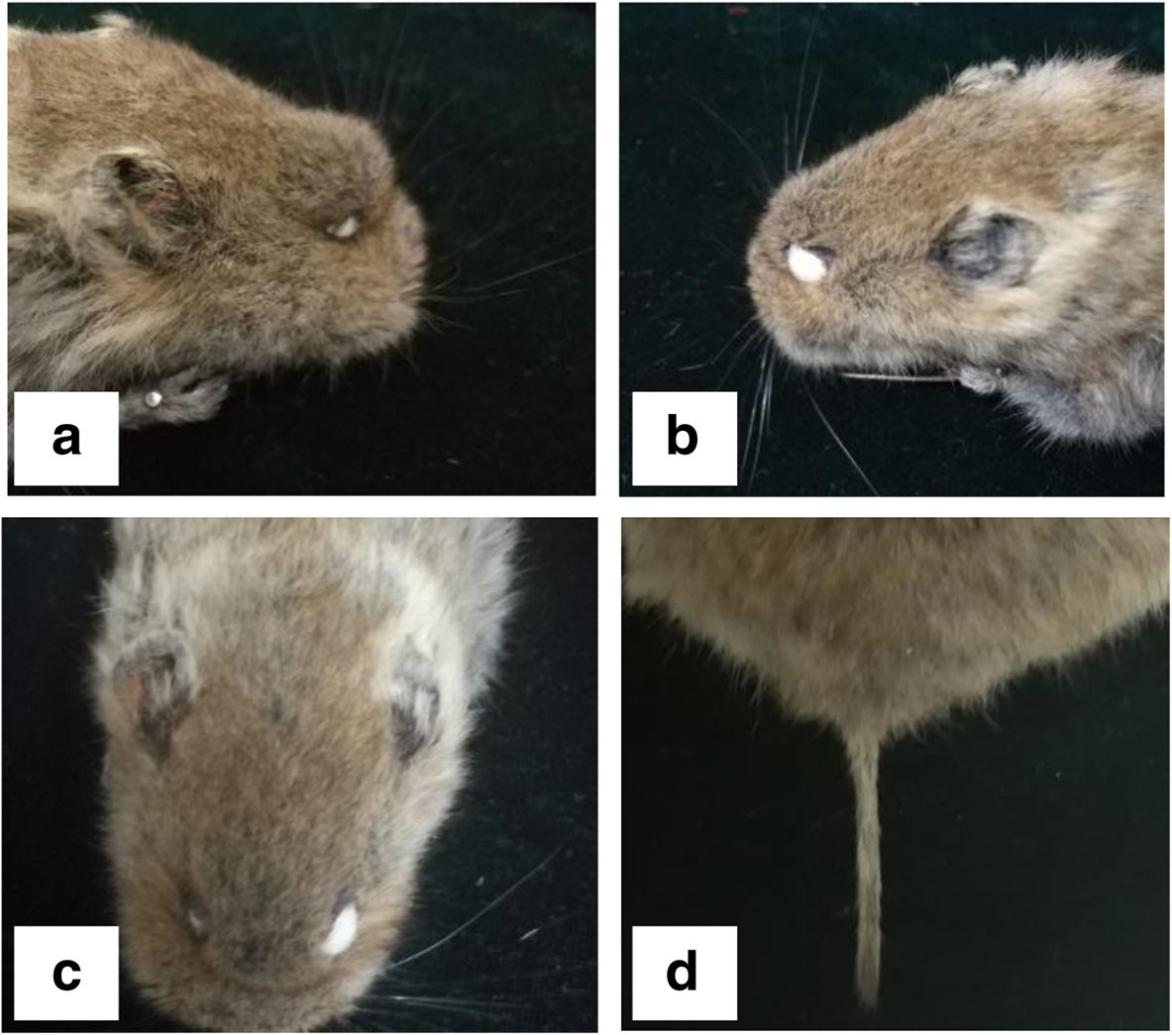
Local features of Qinghai vole: **a** Right lateral view; **b** Left lateral view; **c** Head in full face view; and **d** Tail

The captured voles were of different sizes, presenting body lengths ranging from 6.9 cm to 12.5 cm and weights ranging from 31.5 g to 85.8 g. The animals’ ears were small and round and the tails were short and approximately one third of their body length. All four limbs were short and strong. The paws were powerful for digging holes. The back color was dark brown gray, and the ventral color was grayish yellow. The tails had two different colors. The upper side of the tail proximal to the body was the same color as the back, and the color on the lower side was sandy yellow. The end of the tail was dark brown (Figs. 9 and 10). All of these morphological characteristics combined with other identification data, including the characteristics of the skull and teeth, were the same or similar to the description in the Qinghai Economic Animal Zoography.

**Fig. 11:**
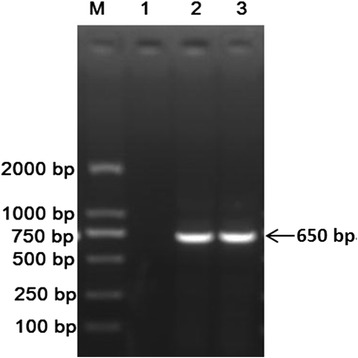
Agarose gel electrophoresis assay of PCR-amplified products of the Qinghai vole’s *cox 1* gene. Lane M: DL 2000 molecular marker; Lane 1: Negative control; Lane 2: Vole’s tail DNA; and Lane 3: Vole’s ear DNA

**Fig. 12:**
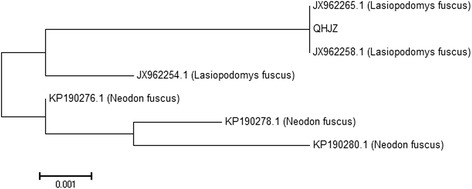
Phylogenetic comparison of the *cox1* sequence from the Qinghai vole with those from the GenBank database. The phylogenetic tree was constructed using the neighbor-joining method and the *p*-distance matrix for nucleotides with the pair-wise gap deletion option. QHJZ represented the Qinghai vole captured in Jigzhi County; JX962254.1, JX962258.1 and JX962265.1 represented *Lasiopodomys fuscus*; and KP190276.1, KP190278.1 and KP190280.1 represented *Neodon fuscus*

DNA segments 650 bp in size from the tissue of Qinghai voles were amplified via PCR (Fig. 11) and used for the phylogenetic analysis along with those of *L. fuscus* and *N. fuscus*. The phylogenetic tree clearly separated into two branches, with one branch belonging to *N. fuscus* and the other belonging to *L. fuscus*. The sequence of a captured Qinghai vole (named QHJZ) was in the *L. fuscus* cluster, which indicated that all of the *E. multilocularis*-infected Qinghai voles were *L. fuscus* (Fig. 12).

## Discussion

The various hosts of *E. multilocularis* are indispensable for the survival and prevalence of this species in the environment and play a key role in the transmission of *E. multilocularis*. Many mammalian species have been identified as definitive or intermediate hosts of *E. multilocularis*, with definitive hosts including red foxes, wolves, domestic dogs, and domestic and wild cats and intermediate hosts including rodents, Eurasian Beavers, brown hare (*Lepus europaeaus*), *Arvicola amphibius*, common vole (*Microtus arvalis*), water vole (*A. terrestris*) and bank vole (*Myodes glareolus*) [26–30, 48]. One important feature of China and its adjacent countries is the large number of mammal species. More than 274 species of mammals have been reported to be candidate intermediate hosts that contribute to *E. multilocularis* transmission [49]. Thus, identifying an intermediate host in rodents is important for determining the transmission pathways of the parasite. Although limited information is available on natural infection of Qinghai voles by *E. multilocularis*, our study demonstrated the presence of *E. multilocularis*-infected Qinghai voles in Jigzhi County of Qinghai Province. In other words, Qinghai voles represent one of the intermediate hosts for *E. multilocularis*, and its role in *E. multilocularis* survival is considerable in Qinghai Province. This information fulfills and enriches the information gap on the life cycle of *E. multilocularis*.

The infection rate of *E. multilocularis* in Qinghai voles in the study region was 22% (11/50). Because human AE infections are prevalent in Qinghai Province, especially in Jigzhi County [38] or neighboring counties, such as Maqing County [20] and Darlag County [34], the large number of Qinghai voles infected with *E. multilocularis* may be highly related to AE infection in humans. However, a positive correlation remains to be demonstrated.

Dogs in the research area, especially Tibetan mastiffs (*Canis lupus familiars*), are definitive hosts of *E. multilocularis*; thus, we should pay more attention to canine hosts. Qinghai voles may be preyed upon by dogs, and during this process, dogs become infected when they ingest protoscoleces-infected Qinghai voles. Previous data showed that the prevalence of *E. multilocularis* infection in dogs was 16% in the southern plateau of Qinghai [31], thus providing evidence of the relationship among infected Qinghai voles, dogs and human AE patients.

Therefore, based on previous data and the results of our study, we can speculate that the definitive hosts, Qinghai voles and humans form a potential life cycle for *E. multilocularis* that is prevalent in Qinghai Province. Canids can prey on infected Qinghai voles and ingest the protoscoleces in the voles’ intestines. Over time, the protoscoleces grow to adult worms and lay eggs in the hosts’ feces, which subsequently contaminate the environment, such as grasslands and water sources. Qinghai voles and humans can be infected by ingesting water or vegetation contaminated with the eggs. After ingestion, the eggs hatch, and then the oncospheres migrate to the liver and develop into metacestode, which produce protoscoleces. To confirm this hypothesis on the life cycle, additional studies should be performed, especially to establish an experimental animal model.

The results in this study provide useful information on the prevalence of *E. multilocularis* and the epidemiological significance of *L. fuscus* as an intermediate host that harbors *E. multilocularis* in the study region. We also present preliminary data on *E. multilocularis* of the Asian genotype. A number of Qinghai voles have been infected with *E. multilocularis* in the study region and thus could act as an important zoonotic reservoir for *E. multilocularis*. However, the epidemiological and clinical significance of our results remain to be further studied. Effective drugs and vaccines are not available for the prevention and control of AE, thus highlighting the importance of our study, which provides insights into the control of AE via the control of Qinghai voles.

## Conclusions

Our study showed that endemic *L. fuscus* can harbor *E. multilocularis* and represents an important intermediate host. This animal species may play a key role in the life cycle and epidemiology of *E. multilocularis* in the Qinghai-Tibetan Plateau of China. These data should be considered for the control of AE via a national campaign program to reduce the high incidence of *E. multilocularis* in the area.

## Additional files

Additional file 1: Multilingual abstracts in the five official working languages of the United Nations. (PDF 698 kb)

Additional file 2: **Figure S1.** Sequence alignment of QH1 to QH11 and AB477011.1 of *E. multilocularis cox* 1 gene. QH1 to QH11: Sequences isolated from Qinghai voles; AB477011.1: Reference sequence downloaded from the GenBank database. (DOCX 44 kb)

## Abbreviations

AE: Alveolar echinococcosis
*Cox 1*: Cytochrome oxidase subunit I
*E. canadensis*: *Echinococcus canadensis*
*E. equinus*: *Echinococcus equinus*
*E. felidis*: *Echinococcus felidis*
*E. granulosus*: *Echinococcus granulosus*
*E. multilocularis*: *Echinococcus multilocularis*
*E. oligarthra*: *Echinococcus oligarthra*
*E. ortleppi*: *Echinococcus ortleppi*
*E. shiquicus*: *Echinococcus shiquicus*
*E. vogeli*: *Echinococcus vogeli*
H-E: Hematoxylin-eosin
*L. fuscus*: *Lasiopodomys fuscus*
TAP: Tibetan Autonomous Prefecture

## References

[1] Nakao M, Lavikainen A, Yanagida T, Ito A (2013). Phylogenetic systematics of the genus *Echinococcus* (Cestoda: Taeniidae). Int J Parasitol.

[2] Maddah G, Abdollahi A, Sharifi-Nooghabi R, Tavassoli A, Rajabi-Mashadi MT, Jabbari-Nooghabi A (2016). Difficulties in the diagnosis and management of alveolar hydatid disease: a case series. Caspian J Int Med.

[3] Wang X, Ding J, Guo X, Zheng Y (2015). Current understandings of molecular biology of *Echinococcus multilocularis*, a pathogen for alveolar echinococcosis in humans- a narrative review article. Iranian J Parasitol.

[4] Gaultier JB, Hot A, Mauservey C, Dumortier J, Coppere B, Ninet J (2009). Granulomatous liver disease as the presenting feature of alveolar echinococcosis in an hepatitis C infected cardiac transplant patient. La Revue de Medecine Interne.

[5] Malikova MS, Frolova Iu V, Raskin VV, Dzemeshkevich AS, Voronina TS, Parshin VD (2012). The simultaneous surgery of heart and echinococcosis under artificial blood circulation. Khirurgiia.

[6] Moro P, Schantz PM (2009). Echinococcosis: a review. Int J Infect Dis.

[7] Massolo A, Liccioli S, Budke C, Klein C (2014). *Echinococcus multilocularis* in North America: the great unknown. Parasite.

[8] Gawor J (2016). Alveolar echinococcosis in Europe and Poland. Threats to humans. Przegl Epidemiol.

[9] Lass A, Szostakowska B, Myjak P, Korzeniewski K (2015). The first detection of *Echinococcus multilocularis* DNA in environmental fruit, vegetable, and mushroom samples using nested PCR. Parasitol Res.

[10] Geramizadeh B, Nikeghbalian S, Malekhosseini SA (2012). Alveolar echinococcosis of the liver: report of three cases from different geographic areas of Iran. Hepat Mon.

[11] Kohansal MH, Nourian A, Bafandeh S (2015). Human cystic echinococcosis in Zanjan Area, Northwest Iran: a retrospective hospital based survey between 2007 and 2013. Iran J Public Health.

[12] Sarkari B, Sadjjadi SM, Beheshtian MM, Aghaee M, Sedaghat F (2010). Human cystic echinococcosis in Yasuj District in Southwest of Iran: an epidemiological study of seroprevalence and surgical cases over a ten-year period. Zoonoses Public Health.

[13] Zibaei M, Azargoon A, Ataie-Khorasgani M, Ghanadi K, Sadjjadi SM (2013). The serological study of cystic echinococcosis and assessment of surgical cases during 5 years (2007-2011) in Khorram Abad, Iran. Niger J Clin Pract.

[14] Benyan AK, Mahdi NK, Abdul-Amir F, Ubaid O (2013). Second reported case of multilocular hydatid disease in Iraq. Qatar Med J.

[15] Al-Attar HK, Al-Irhayim B, Al-Habbal MJ (1983). Alveolar hydatid disease of the liver: first case report from man in Iraq. Ann Trop Med Parasitol.

[16] Feng X, Qi X, Yang L, Duan X, Fang B, Gongsang Q (2015). Human cystic and alveolar echinococcosis in the Tibet Autonomous Region (TAR), China. J Helminthol.

[17] Han J, Bao G, Zhang D, Gao P, Wu T, Craig P (2015). A newly discovered epidemic area of echinococcus multilocularis in West Gansu Province in China. PLoS One.

[18] Li T, Chen X, Zhen R, Qiu J, Qiu D, Xiao N (2010). Widespread co-endemicity of human cystic and alveolar echinococcosis on the eastern Tibetan plateau, Northwest Sichuan/Southeast Qinghai, China. Acta Trop.

[19] Ma YA, Shang WJ (2015). Endemic situation of echinococcosis in Gannan Tibetan Autonomous Prefecture. Zhongguo Ji Sheng Chong Xue Yu Ji Sheng Chong Bing Za Zhi..

[20] Ma X, Wang H, Han XM, Zhang JX, Liu YF, Zhao YM (2015). Survey on echinococcosis in Maqing County of Qinghai Province. Zhongguo Ji Sheng Chong Xue Yu Ji Sheng Chong Bing Za Zhi..

[21] Jeong JS, Han SY, Kim YH, Sako Y, Yanagida T, Ito A (2013). Serological and molecular characteristics of the first Korean case of *Echinococcus multilocularis*. Korean J Parasitol.

[22] Nonaka N, Kamiya M, Oku Y (2009). A vague understanding of the biology and epidemiology of echinococcosis by dog owners in Hokkaido, an endemic island for *Echinococcus multilocularis* in Japan. J Vet Med Sci.

[23] Jiang W, Liu N, Zhang G, Renqing P, Xie F, Li T (2012). Specific detection of *Echinococcus* spp. from the Tibetan fox (Vulpes ferrilata) and the red fox (*V. vulpes*) using copro-DNA PCR analysis. Parasitol Res.

[24] Romig T, Deplazes P, Jenkins D, Giraudoux P, Massolo A, Craig PS (2017). Ecology and life cycle patterns of *Echinococcus* species. Adv Parasitol.

[25] Giraudoux P, Raoul F, Afonso E, Ziadinov I, Yang Y, Li L (2013). Transmission ecosystems of *Echinococcus multilocularis* in China and Central Asia. Parasitology.

[26] Miller AL, Olsson GE, Walburg MR, Sollenberg S, Skarin M, Ley C (2016). First identification of *Echinococcus multilocularis* in rodent intermediate hosts in Sweden. Int J Parasitol Parasite Wildl.

[27] Nakao M, Sako Y, Yokoyama N, Fukunaga M, Ito A (2000). Mitochondrial genetic code in cestodes. Mol Biochem Parasitol.

[28] Viel JF, Giraudoux P, Abrial V, Bresson-Hadni S (1999). Water vole (*Arvicola terrestris scherman*) density as risk factor for human alveolar echinococcosis. Am J Trop Med Hyg..

[29] Woolsey ID, Bune NE, Jensen PM, Deplazes P, Kapel CM (2015). *Echinococcus multilocularis* infection in the field vole (*Microtus agrestis*): an ecological model for studies on transmission dynamics. Parasitol Res.

[30] Campbell-Palmer R, Del Pozo J, Gottstein B, Girling S, Cracknell J, Schwab G (2015). *Echinococcus multilocularis* detection in live Eurasian beavers (*Castor fiber*) using a combination of laparoscopy and abdominal ultrasound under field conditions. PLoS One.

[31] Cai HX, Guan YY, Wang H, Wu WP, Han XM, Ma X (2012). Geographical distribution of echinococcosis among children in Qinghai Province. Zhongguo Ji Sheng Chong Xue Yu Ji Sheng Chong Bing Za Zhi..

[32] Fu Q, Han XM, Wang LY, Sangba DY, Ma X, Wang YS (2010). Investigation on epidemic status of echinococcosis in pastoral villages of Chengduo county, Qinghai. Zhonghua Liu Xing Bing Xue Za Zhi.

[33] Giraudoux P, Raoul F, Pleydell D, Li T, Han X, Qiu J (2013). Drivers of *Echinococcus multilocularis* transmission in China: small mammal diversity, landscape or climate?. PLoS Negl Trop Dis.

[34] Han XM, Wang H, Cai HX, Ma X, Liu YF, Wei BH (2009). Epidemiological survey on echinococcosis in Darlag County of Qinghai Province. Zhongguo Ji Sheng Chong Xue Yu Ji Sheng Chong Bing Za Zhi.

[35] Luo A, Wang H, Li JQ, Wu HS, Yang F, Fang PQ (2014). Epidemic factors and control of hepatic echinococcosis in Qinghai province. Hua Zhong Ke Ji Da Xue Xue Bao Yi Xue Ying De Wen Ban.

[36] Wang Q, Huang Y, Huang L, Yu W, He W, Zhong B (2014). Review of risk factors for human echinococcosis prevalence on the Qinghai-Tibet plateau, China: a prospective for control options. Infect Dis Poverty.

[37] Wu XH, Wang H, Zhang JX, Ma X, Liu YF, Han XM (2007). An epidemiological survey on echinococcosis in Zhiduo County of Qinghai Province. Zhongguo Ji Sheng Chong Xue Yu Ji Sheng Chong Bing Za Zhi.

[38] Wu XH, Wang H, Kawanaka M, Morishima Y, Ma X, Liu P (2007). Epidemiologic survey and studies on echinococcosis in humans in Jiuzhi County of Qinghai Province. Chin J Zoonoses.

[39] Xiao N, Nakao M, Qiu J, Budke CM, Giraudoux P, Craig PS (2006). Dual infection of animal hosts with different *Echinococcus* species in the eastern Qinghai-Tibet plateau region of China. Am J Trop Med Hyg.

[40] Hoffmann RS (1996). Noteworthy shrews and voles from the Xizang-Qinghai Plateau.

[41] Li D, Wang Z, Wu Y, Zheng C, Huang Y, Cai G (1989). Qinghai Economic Animal Zoograpy.

[42] Smith AT, Yan X (2008). A guide to the mammals of China.

[43] Wang S, Xie Y (2004). China species red list, volume 1: red list.

[44] Zhang Z (2004). Qinghai Dili.

[45] Ma J, Wang H, Lin G, Craig PS, Ito A, Cai Z (2012). Molecular identification of *Echinococcus* species from eastern and southern Qinghai, China, based on the mitochondrial cox1 gene. Parasitol Res.

[46] Northwest Institute of Plateau Biology, Chinese Academy of Sciences (1989). Qinghai economic animal zoography.

[47] Ivanova NV, DeWaard JR (2006). Heber tPDN. An inexpensive, automation-friendly protocol for recovering high-quality DNA. Mol Ecol Notes.

[48] Chaignat V, Boujon P, Frey CF, Hentrich B, Muller N, Gottstein B (2015). The brown hare (*Lepus europaeus*) as a novel intermediate host for *Echinococcus multilocularis* in Europe. Parasitol Res.

[49] Xie Y, MacKinnon J, Li D (2004). Study on biogeographical divisions of China. Biodivers Conserv.

