# Supplementary material for: Lasiopodomys fuscus as an important intermediate host for Echinococcus multilocularis: isolation and phylogenetic identification of the parasite
Source: Infect Dis Poverty. 2018 Mar 31;7:27. doi: 10.1186/s40249-018-0409-4 (PMC5878421; doi:10.1186/s40249-018-0409-4)
Supplement: Supplementary file 1 — Multilingual abstracts in the five official working languages of the United Nations. (PDF 698 kb) [file 40249_2018_409_MOESM1_ESM.pdf]

Translation of the abstract into the six official working languages of the United Nations

القوارض البُنِّية كمضيف وسيط هام لـ الديدان الشريطية متعددة الخلايا : عزل وتحديد تطور الطفيل

تشى جانغ تساى، شيوى مين هان، يونغ هاي يانغ، شيويه يونغ تشانغ، لى تشينغ ما، باناجيوتيس كارانيس، يونغ هاو هو

الملخص

الخلفية: تتسبب الديدان الشريطية متعددة الخلايا في الإصابة بمرض المشوكات الحويصلي (AE) المنتشر على نطاق واسع في مقاطعة تشينغهاي الصينية، حيث تم تحديد عدد من الأنواع المختلفة كمضيفين. ومع ذلك، تتوفر معلومات محدودة عن قوارض تشينغهاي (القوارض البنية)، المتوطنة بصورة كبيرة في مقاطعة تشينغهاي وقد تمثل مضيف وسيط محتمل لـ للديدان الشريطية متعددة الخلايا. وبالتالي، يمكن أن تساعدالقوارض البنية في توطن مرض المشوكات الحويصلي AE في المنطقة.

الطرق: تم اصطياد خمسين من قوراض تشينغهاي من منطقة جيجزي في مقاطعة تشينغهاي للتعرف السريري على عدوى الديدان الشريطية متعددة الخلايا عن طريق الفحص التشريحي. تم جمع السائل العدارى من حويصلات الكبد في القوارض المشتبه بها وتعرضت للفحص المجهري ومقايسة تفاعل البلمرة المتسلسل (PCR) على أساس الجين الشريطي لـ cox 1 . تم تسلسل شرائح PCR المتضخمة لتحليل التطور الوراثي. وتم تحديد قوارض تشينغهاي المصابة بشكل مورفولوجي بالديدان الشريطية متعددة الخلايا وإخضاعها لتحليل التطور الوراثي لتأكيد هوياتها.

النتائج: كانت سبعة عشر من قوارض تشينغهاي الـ 50 مصابة بالديدان الشريطية متعددة الخلايا بعدوى حويصلات في الكبد. وقد أظهرأحد عشر من أصل 17 من قوارض تشينغهاي الإصابةبالديدان الشريطية متعددة الخلايا ، والتي تم اكتشافها بواسطة PCR والتسلسل. وأظهر تحليل التطور الوراثي أن جميع العينات الإيجابية الـ 11 تنتمي إلى الديدان الشريطية متعددة الخلايا ذات التركيب الوراثي الآسيوي. وأكد التعريف المورفولوجي والتحليل الوراثي لـ قوارض تشينغهاي المصابة بالديدان الشريطية متعددة الخلايا أن جميع الحيوانات التي تم اصطيادها كانت قوارض بنية .

الاستنتاجات: يمكن أن تصاب القوارض البنية بـ بالديدان الشريطية متعددة الخلايا وتلعب دورًا محتملاً في دورة الحياة وعلم الأوبئة للديدان الشريطية متعددة الخلايا في هضبة تشينغهاي التبتية في الصين.

Translated from English version into Arabic by Free Bird, proofread by Noor Alquraishi, through

TRANSLATORS
WITHOUT BORDERS

青海田鼠（*Lasiopodomys fuscus*）作为多房棘球绦虫的一种重要的中间宿主：多房棘球蚴绦虫的分离及系统发育鉴定

蔡其刚，韩秀敏，杨永海，张学勇，马利青，帕纳约蒂斯 卡纳尼斯，胡永浩

摘要

引言：多房棘球绦虫可导致泡型包虫病的发生并在中国青海省广泛流行。在此，许多不同种动物已被确定为其宿主。然而，却鲜有青海田鼠(*Lasiopodomys fuscus*)作为其宿主的相关报道。青海田鼠仅生活在青海省，其可能作为多房棘球绦虫潜在的中间宿主。因此，青海田鼠对于泡型包虫病在该地区的流行可能起到一定的促进作用。

方法：从青海省久治县共捕获 50 只田鼠，首先对其开展形态学鉴定和系统发育分析，确认其物种身份。其次，对其解剖观察进行多房棘球绦虫的临床鉴定。然后，从疑似多房棘球蚴绦虫感

染的田鼠肝脏囊泡中收集囊液进行镜检。最后，开展基于条形码基因 *cox1* 基因的 PCR 鉴定，对 PCR 扩增的基因片段序列测定后进行系统发育分析。

结果：经形态学鉴定和系统进化分析确定，所捕获的 50 只田鼠均为青海田鼠（*L. fuscus*）。其中在 17 只青海田鼠的肝脏上发现疑似多房棘球绦虫感染的囊泡。通过 PCR 和序列测定分析，确认其中 11 只青海田鼠感染了多房棘球绦虫。系统发育分析显示，这 11 株多房棘球绦虫均属于亚洲型多房棘球绦虫。

结论：青海田鼠可以被多房棘球绦虫感染，且其在多房棘球绦虫的生活史及中国青藏高原上的流行方面起到潜在性的作用。

Translated from English version into Chinese by Qi-gang Cai, edited by Zhi-liang Wang (China Animal Health And Epidemiology Center)

Lasiopodomys fuscus en tant qu'hôte intermédiaire important pour _Echinococcus multilocularis_: isolement et identification phylogénétique du parasite

Qi-Gang Cai, Xiu-Min Han, Yong-Hai Yang, Xue-Yong Zhang, Li-Qing Ma, Panagiotis Karanis, Yong-Hao Hu

RÉSUMÉ

Contexte: *Echinococcus multilocularis* est à l'origine de la maladie parasitaire échinococcose alvéolaire (EA) et est largement répandu dans la province de Qinghai en Chine, où différentes espèces ont été identifiées en tant qu'hôtes. Il n'existe toutefois que peu d'information sur le campagnol de plateau (*Lasiopodomys fuscus*), dont la présence dans la province de Qinghai est hyper-endémique et susceptible de constituer un hôte intermédiaire potentiel de l'*échinococcose alvéolaire*. *Lasiopodomys fuscus* pourrait ainsi contribuer à ce que cette zone devienne un foyer endémique de l'échinococcose alvéolaire.

Méthodes: Cinquante campagnols de plateau ont été capturés dans le xian de Jigzhi au sein de la province de Qinghai afin de procéder à l'identification clinique de l'infection à *Echinococcus multilocularis* à l'aide d'examens de leurs anatomies. Des ponctions de liquide hydatique sur des alvéoles dans les foies de campagnols suspects ont permis de procéder à un examen microscopique et à un test PCR sur la base du code-barres génétique du *Cox 1*. Le séquençage des segments amplifiés par PCR a ensuite permis une analyse phylogénétique. Les campagnols de plateau infectés par *Echinococcus multilocularis* ont fait l'objet d'une identification morphologique, suivie d'une analyse phylogénétique pour confirmer leurs identités.

Résultats: Dix-sept des 50 campagnols de plateau étaient porteurs d'alvéoles apparemment infectées par*Echinococcus multilocularis* dans leurs foies. Onze des 17 campagnols de plateau présentaient une infection à *Echinococcus multilocularis*, qui a été détectée par PCR et séquençage. L'analyse phylogénétique a révélé que la totalité des 11 souches positives appartenait au génotype asiatique *Echinococcus multilocularis*. Une identification morphologique et une analyse phylogénétique des campagnols de plateau infectés par *Echinococcus multilocularis* ont confirmé que tous les animaux capturés étaient des *Lasiopodomys fuscus*.

Conclusions: *L. fuscus* peut être infecté par *E. multilocularis* et jouer un rôle potentiel dans le cycle biologique et l'épidémiologie d'*E. Multilocularis* dans le plateau Qinghai-Tibet de la Chine.

Translated from English version into French by Elisa Griffin, proofread by Vero Marie, through

TRANSLATORS WITHOUT BORDERS

Lasiopodomys fuscus как важный промежуточный хозяин _Echinococcus multilocularis_: изолирование и филогенетическая идентификация паразита

Qi-Gang Cai, Xiu-Min Han, Yong-Hai Yang, Xue-Yong Zhang, Li-Qing Ma, Panagiotis Karanis, Yong-Hao Hu

КРАТКОЕ ОПИСАНИЕ

Справочная информация: *Echinococcus multilocularis* вызывает альвеолярный эхинококкоз (АЭ) и широко распространен в Провинции Цинхай (Китай), где в качестве хозяев были определены несколько разных видов. Однако, информация о цинхайских полевках (*Lasiopodomys fuscus*), широко эндемичных в Провинции Цинхай, которые могут быть потенциальными хозяевами *E. multilocularis*, ограничена. Таким образом, *L. fuscus* может способствовать эндемичности АЭ в этом районе.

Методы: В целях клинической идентификации инфекции *E. multilocularis* посредством анатомического обследования были пойманы пятьдесят цинхайских полевок в округе Джигджи Провинции Цинхай. Эхинококковые жидкости были собраны из печеночных пузырей предположительно больных полевок и подвергнуты микроскопическому обследованию и ПЦР-анализу на основании ДНК-штрихкодирования *cox 1*. Для филогенетического анализа были упорядочены амплифицированные методом ПЦР сегменты. Цинхайские полевки, инфицированные*E. multilocularis*, были морфологически выделены и подвергнуты филогенетическому анализу с целью подтверждения их идентичностей.

Результаты: У семнадцати из 50 цинхайских полевок в печени были найдены пузырьки, похожие на инфекцию *E. multilocularis*. У одиннадцати из 17 цинхайских полевок была выявлена инфекция *E. multilocularis* , обнаруженная посредством ПЦР и секвенирования . Филогенетический анализ показал, что все 11 положительных образцов относились к азиатскому генотипу*E. multilocularis*. Морфологическая идентификация и филогенетический анализ цинхайских полевок, инфицированных *E. multilocularis,* подтвердили, что все пойманные животные были *L. fuscus*.

Выводы: *L. fuscus* могут быть инфицированы *E. multilocularis,* и, предположительно,участвовать в жизненном цикле и эпидемиологии *E. multilocularis* на Цинхай-Тибетском нагорье Китая.

Translated from English version into Russian by Tatiana Kary, proofread by Galina Dmitrieva, through

***Lasiopodomys fuscus* como un importante huésped intermediario para *Echinococcus multilocularis*: aislamiento e identificación filogenética del parásito**

Qi-Gang Cai, Xiu-Min Han, Yong-Hai Yang, Xue-Yong Zhang, Li-Qing Ma, Panagiotis Karanis, Yong-Hao Hu

RESUMEN

Contexto: la*Echinococcus multilocularis* provoca equinococosis alveolar (EA) y es prevalente en la provincia de Qinghai, China, en donde se han identificado varias especies diferentes como sus huéspedes. Sin embargo, hay muy poca información acerca del ratón de campo de Qinghai (*Lasiopodomys fuscus*), que es hiperendémico en la provincia de Qinghai y podría ser un huésped intermediario potencial de *E. multilocularis*. Por tanto, el *L. fuscus* podría contribuir al desarrollo de la EA como una endemia en la zona.

Métodos: Se capturaron cincuenta ratones de campo de Qinghai del condado de Jigzhi, provincia de Qinghai, para la identificación clínica de infecciones por *E. multilocularis* mediante un examen anatómico. Se extrajo líquido hidático de vesículas de los hígados de los posibles huéspedes, y se lo sometió a un examen microscópico y a un análisis de PCR basándose en el gen de código de barras de *cox 1*. Los segmentos con PCR amplificado luego se secuenciaron para realizar un análisis filogenético. Se identificó morfológicamente a los ratones de campo de Qinghai infectados con *E. multilocularis* y se los sometió a un análisis filogenético para confirmar su identidad.

Resultados: Diecisiete de los cincuenta ratones de campo de Qinghai tenían vesículas con infecciones similares a *E. multilocularis* en el hígado. Once de los diecisiete ratones de campo de Qinghai presentaban infecciones por *E. multilocularis*, que se detectaron mediante análisis de PCR y secuenciación. El análisis filogenético demostró que las 11 muestras positivas pertenecían al genotipo asiático de *E. multilocularis*. Una identificación morfológica y un análisis filogenético de los ratones de campo de Qinghai infectados con *E. multilocularis* confirmaron que todos los animales capturados eran *L. fuscus*.

Conclusiones: El *L. fuscus* puede infectarse con *E. multilocularis* y desempeña un papel potencial en el ciclo de vida del *E. multilocularis* y su epidemiología en la meseta Tibetana-Qinghai de China.

Translated from English version into Spanish by Jimena, proofread by María Emilia Meini, through
