# Supplementary material for: Lasiopodomys fuscus as an important intermediate host for Echinococcus multilocularis: isolation and phylogenetic identification of the parasite
Source: Infect Dis Poverty. 2018 Mar 31;7:27. doi: 10.1186/s40249-018-0409-4 (PMC5878421; doi:10.1186/s40249-018-0409-4)
Supplement: Supplementary file 2 — Figure S1. Sequence alignment of QH1 to QH11 and AB477011.1 of E. multilocularis cox 1 gene. QH1 to QH11: Sequences isolated from Qinghai voles; AB477011.1: Reference sequence downloaded from the GenBank database. (DOCX 44 kb) [file 40249_2018_409_MOESM2_ESM.docx]

Additional file 1

1. Alignment of the 11 sequences with the reference sequence (GenBank Accession No: AB477011.1)

The results showed that 47 nucleotide sites were different from those of the reference sequence. The different nucleotide sites exhibited 4 characteristics: the sequences of QH7 and QH4 showed 13 variable nucleotide sites (site 2 to 123); the sequences of QH1, QH6 and QH3 showed 30 different nucleotides sites (site 697 to 788), including the sequence of QH8 at site 770; the nucleotide at site 124 of all 11 sequences except QH8 was mutated from C to T; and the sequences of QH8, QH5 and QH7 shared the last 3 nucleotides at sites 269, 271 and 294.

**
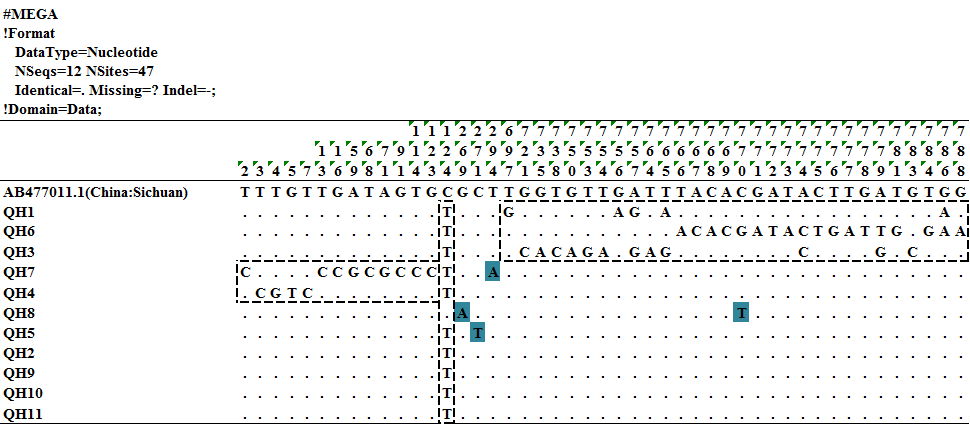
**

**Figure S1** Sequence alignment of QH1 to QH11 and AB477011.1 of *E. multilocularis cox* 1 gene. QH1 to QH11: Sequences isolated from Qinghai voles; AB477011.1: Reference sequence downloaded from the GenBank database

2. The accepted accession numbers submitted in GenBank database of the 11 isolated *cox 1* genes

**Table S1**: Accepted accession numbers compared with the 11 isolated *cox 1* genes

| **Isolated sequences name** | **GenBank accession number** |
| --- | --- |
| QH1 | KY062624 |
| QH2 | KY062625 |
| QH3 | KY062626 |
| QH4 | KY062627 |
| QH5 | KY062628 |
| QH6 | KY062629 |
| QH7 | KY062630 |
| QH8 | KY062631 |
| QH9 | KY062632 |
| QH10 | KY062633 |
| QH11 | KY062634 |
